# The effect of payment and incentives on motivation and focus of community health workers: five case studies from low- and middle-income countries

**DOI:** 10.1186/s12960-015-0051-1

**Published:** 2015-07-14

**Authors:** Debra Singh, Joel Negin, Michael Otim, Christopher Garimoi Orach, Robert Cumming

**Affiliations:** Research and Collaboration, Kimanya-Ngeyo Foundation for Science and Education, Jinja, Uganda; School of Public Health, University of Sydney, Sydney, Australia; School of Allied Health | Faculty of Health Sciences, Australian Catholic University, Sydney, Australia; School of Public Health, Makerere University, Kampala, Uganda

**Keywords:** Community health workers, Payment, Volunteerism, Remuneration, Motivation, Altruistic capital

## Abstract

**Introduction:**

Community health workers (CHWs) have been proposed as a means for bridging gaps in healthcare delivery in rural communities. Recent CHW programmes have been shown to improve child and neonatal health outcomes, and it is increasingly being suggested that paid CHWs become an integral part of health systems. Remuneration of CHWs can potentially effect their motivation and focus. Broadly, programmes follow a social, monetary or mixed market approach to remuneration. Conscious understanding of the differences, and of what each has to offer, is important in selecting the most appropriate approach according to the context.

**Case descriptions:**

The objective of this review is to identify and examine different remuneration models of CHWs that have been utilized in large-scale sustained programmes to gain insight into the effect that remuneration has on the motivation and focus of CHWs. A MEDLINE search using Ovid SP was undertaken and data collected from secondary sources about CHW programmes in Iran, Ethiopia, India, Bangladesh and Nepal. Five main approaches were identified: part-time volunteer CHWs without regular financial incentives, volunteers that sell health-related merchandise, volunteers with financial incentives, paid full-time CHWs and a mixed model of paid and volunteer CHWs.

**Discussion and evaluation:**

Both volunteer and remunerated CHWs are potentially effective and can bring something to the health arena that the other may not. For example, well-trained, supervised volunteers and full-time CHWs who receive regular payment, or a combination of both, are more likely to engage the community in grass-roots health-related empowerment. Programmes that utilize minimal economic incentives to part-time CHWs tend to limit their focus, with financially incentivized activities becoming central. They can, however, improve outcomes in well-circumscribed areas. In order to maintain benefits from different approaches, there is a need to distinguish between CHWs that are trained and remunerated to be a part of an existing health system and those who, with little training, take on roles and are motivated by a range of contextual factors. Governments and planners can benefit from understanding the programme that can best be supported in their communities, thereby maximizing motivation and effectiveness.

## Background

### Community health workers

Community health workers (CHWs) have been defined as lay persons who have received some training to deliver healthcare services but are not professionals [1]. Broadly, CHWs can be viewed as individuals whose primary role is to extend the reach of the existing mainstream health system [2] or playing a broader role by serving as cultural mediators or change agents by facilitating grass-roots community engagement to improve health outcomes [2-4]. They have been proposed as a means of bridging the gap in the current health systems in many low- and middle-income countries (LMIC) but have also shown promise, particularly with chronic disease management in high-income countries [5,6]. Recent studies have shown that when CHWs are well trained and supervised they can positively impact outcomes in maternal and child health:

“CHW’s are most effective when supported by a clinically skilled health workforce, particularly for maternal health, and deployed within the context of an appropriately financed primary health care system. However, CHW’s have also notably proven crucial in settings where the overall primary health care system is weak, particularly in improving child and neonatal health. They also represent a strategic solution to address the growing realization that the shortages of highly skilled health workers will not meet the growing demand of the rural population. As a result, the need to systematically and professionally train lay community members to be part of the health workforce has emerged not simply as a stop-gap measure, but as a core component of primary health care systems in low-resource settings.” [7].

In the 1970s and 1980s, there was concern about CHW effectiveness due to high-attrition rates, particularly when smaller programmes attempted to go to scale [7]; however, some long-standing and more recently initiated large-scale programmes have shown promise [7-10]. Political will and commitment have been evident in these successful larger scale programmes, each of which has taken a somewhat different approach to training, supervising and remunerating CHWs [11-14]. Based on the experiences of some of these countries, it is increasingly being suggested that CHWs be paid and fully integrated into health systems [7]. Integration suggests clearly delineated responsibilities within the health system, fair remuneration and, in some cases, the possibility of a career path [7].

### Debate on remuneration

While CHW programmes worldwide have taken a variety of approaches to payment, many have operated with relatively low budgets and engaged volunteers. Lack of remuneration has been an often-stated cause of poor retention of CHWs [3,8]. While this may be a contributing factor, the desire to give of one’s time is determined by a number of issues. Volunteers in wealthier countries generally have other incomes, such as pensions, spousal income or part-time employment, and find it a meaningful way to occupy their time or make a contribution to society. In lower socio-economic environments – despite low-education levels – building capacity to be able to contribute to one’s community is an important consideration. A recent study in Uganda showed that CHWs while wishing to be remunerated felt that issues such as community recognition and development of new skills and knowledge [15] outweighed the disadvantage of a lack of funding. Religious beliefs and spirituality seem important in all contexts [16]. The time one has to volunteer is, however, generally determined by one’s income source. A female farmer in Africa, for example, will have limited time to volunteer compared to a pensioner in a higher income country.

Cherrington et al. [17] note that there are those who feel that CHWs deserve to be paid and those who argue that receiving a wage is contrary to the very nature of lay advising but that there are legitimate arguments for utilizing both the paid and volunteer models, depending on the context, community needs and programme goals. To avoid the loss of the important characteristics that volunteers bring, it may be necessary to redefine what a “CHW” is, as some take on higher levels of training and a greatly expanded role in existing health systems.

### Behavioural economics and remuneration of CHWs

Behavioural economics, which has gained attention in the past decade among economists, psychologists and social scientists, may provide some insights into CHW remuneration, focus and motivation. While not completely rejecting neoclassical economics, behavioural economics does challenge the assumptions on which it is built. Pink [18] and Dawney and Shah [19] delineate a number of motivators that may be important and are responsive to “more complex aspects of human nature” than are addressed by neoclassical economics. These include autonomy, mastery, purpose and connectedness (see Table 1). Additionally, research emerging from the Harvard Business School [20] suggests that behavioural economics, and particularly “altruistic capital”, may have an important role in understanding the motivation of CHWs. Altruistic capital, as defined by this group, means that every individual, in varying degrees, has within them an intrinsic desire to serve others.

**Table 1:** **Motivators: according to behavioural economics theory**

**Behavioural economists perspectives on motivation: internal and external**	
**Internal motivators (Pink [** 9 **])**	**External motivators (Heyman and Ariely [** 11 **])**
Autonomy: acting with choice, does not exclude interdependence with others. The opposite of autonomy is control. Control leads to compliance while autonomy leads to engagement.	Monetary markets: when payments were given in the form of cash, effort seemed to stem from reciprocation and was sensitive to the magnitude of the payment.
Mastery: the desire to be better and better at something that matters.	Social markets and gifts: when payments were given in the form of gifts or when payments were not mentioned, effort seemed to stem from altruistic motives and was largely insensitive to the magnitude of the payment.
Purpose and connectedness: those who work in service for a greater purpose than themselves can achieve more than those that do not.	Mixed markets: the mere mention of monetary payment was sufficient to switch the perceived relationship from a social-market relationship to a money-market relationship.

Heyman and Ariely [11] draw attention to a distinction between monetary markets and social markets (see Table 1), hypothesizing that “monetary markets are highly sensitive to the magnitude of compensation, whereas social markets are not”. This perspective sheds light on the well-established observation that people sometimes expend more effort in exchange for no payment (a social market) than they expend when they receive payment (a monetary market). They conclude that, “mixed markets (markets that include aspects of both social and monetary markets) more closely resemble monetary than social markets”.

Incentives for health workers have also been proposed as a means of improving health outcomes and have been utilized in a number of settings, with varying degrees of success. When discussing incentives for different cadres of health professionals, Hongoro and McPake [21] acknowledge the difficulties in designing incentives for health workers that are financially based in that they may undermine the “ethos” of public service that motivates them. Gifts and community recognition are considered to be within the domain of social markets and appear to be important particularly for the motivation of both employed individuals and volunteers, as they are considered to reinforce rather than undermine efforts [11].

At a time when considerable attention is being paid to how CHWs can be best utilized and integrated into the health system, it seems necessary to try to distinguish between approaches and motivators. The objective of this review is to identify and examine different remuneration models of CHWs that have been utilized in large-scale sustained programmes to gain insight into the effect that remuneration has on the motivation and focus of the CHWs. Five models of CHW remuneration will be considered as described in the “Methods” section below.

## Case descriptions

### Methods

In order to classify and examine different remuneration models of CHWs, a MEDLINE search using Ovid SP was conducted between September 2013 and May 2014 and citation searches were also carried out. Search terms included CHWs, remuneration, volunteers, behavioural economics and health, motivation, payment and altruistic capital. An initial 18 review and related articles, books and theses were also identified, and the case studies selected. A further 34 research articles (minimum of 5 per programme) and descriptions of the specific CHW programmes were also identified by citation snowballing from the original journal articles. Selection criteria for including programmes as case studies were those that had been sustained for at least 5 years and had a retention rate of 85% or more – this was taken as an indicator of the sustainability of the programme. A multiple case study approach [22] was selected rather than a systematic review because of the lack of uniformity in CHW programmes worldwide. All of the programmes selected had broad experience, having trained at least 30 000 CHWs each. Programmes included had a minimum of five relevant publications available for review. A programme was not included if it trained fewer than 30 000 CHWs or was not sustained.

This proposed inquiry aimed to identify all relevant literature pertaining to the remuneration issues of selected CHW programmes. The authors believed that a combination of qualitative, quantitative and mixed methods studies presented more enhanced insights and addressed the research question. Hence, a mixed studies review (MSR) was conducted that allowed a search strategy including diverse designs.

The case studies provided a variety of documented approaches to remuneration including (a) part-time volunteers, working limited hours without regular financial incentives: the Female Community Health Volunteer (FCHV) programme from Nepal; (b) volunteers that sell health merchandise: Bangladesh Rural Advancement Committee (BRAC) Community Health Volunteer (CHV) programme in Bangladesh; (c) volunteers with financial incentives: the Accredited Social Health Activist (ASHA) programme in India; (d) paid full-time CHWs: the CHWs (Behvarzs) in Iran; and (e) both full-time and volunteer CHWs working together: Health Extension Programs (HEPs) in Ethiopia. Not all large-scale CHW programmes have been included due to space limitations. For example, the Brazilian government’s Community Health Agent programme and Pakistan’s Lady Health Worker programme [10] are examples of well-known and documented paid full-time CHW programmes [7]. The programme in Iran was selected because it has not received as wide an exposure despite its success. The others that were included are well known but have very distinct approaches to remuneration, which lent themselves to a useful contrast. Similarly, the multiple approaches taken in the developed world/United States context, while of interest, is beyond the scope of this paper [5,6].

Each of the case studies addressed three central questions – (a) What is the history and description of the programme? (b) What has been the impact of the programme? and (c) What have been the reported effects of the remuneration model? An initial case study was prepared and compared with the set of questions that were to be answered. Further literature searches were undertaken until the questions were answered or it was evident that the answers had not been published.

Table 2 summarizes the five CHW case studies considered in this paper.

**Table 2:** **Summary of CHW programmes in the case studies**

	**Iran Behvarzs**	**Ethiopia Health Extension Workers – blended programme of full time and part time**	**India Accredited Social Health Activists (ASHAs)**	**Bangladesh Community Health Volunteers (CHVs)**	**Nepal Female Community Health Volunteers (FCHVs)**
Behavioural Economics Model	Monetary market: paid for full-time work	Monetary market: paid for full-time work	Mixed market: incentives	Mixed market: selling of health commodities	Social market: volunteers
Working hours	Full time	Full time	Part time	Part time 15–20 h	Part-time 5–10 h per week
Current number	31 000	38 000	820 000	80 000	48 000
Minimum education level	Completed high school	Grade 10	Grade 8	Some years of school	Literate if possible
Ratio	1:1500	1:2500	1:1000	1:1500	1:400
Training and supervision	2 years full time, refresher courses and monthly meetings	1 year full time	23 days then attend weekly meeting	Initial 21 days then supervised 2–3 times a month	Initial 15 days then refresher once a month
Impact	Reduced IMR, MMR, increased life expectancy	Decreased MMR, IMR, increased family planning, clean water, HIV tests	Increased facility-based deliveries, decreased MMR and neonatal mortality	NA	Decreased IMR under-5 mortality and morbidity
Retention	Required to work for 4 years in government service	93.5–99% over a 1- to 6-year period	NA	84–89%	85% over 5 years
Advantages	High-retention rates, high-quality service in rural areas	High coverage, allows for extension of health services and community engagement	Increased coverage for specific health interventions	Marginalized women have a chance to earn small incomes and to engage in health	High levels of community good will and support
Disadvantages	High cost, long training period before starting to work	High cost, long training period before starting to work	Focus on incentivized health interventions	Focus on selling health commodities could distract from health issues	Other commitments may create less time for the field work

### Case studies

#### Part-time volunteer CHWs without financial incentives: Female Community Health Volunteer programme in Nepal

##### History and description

The programme in Nepal is a volunteer-based programme that has been operating since 1988. There are 48 000 FCHVs that generally volunteer 5 h per week. The FCHVs are women, at least 20 years of age and literate where possible. If she is not literate, she should be willing to attend literacy classes. Preference is given to women who have been involved in social action previously and who have one or two children [9]. There is generally one FCHV for a population of 400 people, with smaller ratios of FCHV and people in hills and mountains. The FCHVs receive an initial 15 days of training with refresher courses every 6 months. The training is provided by health assistants, assistant nurse midwives and auxiliary health workers under the direction of the district health officer. The FCHVs develop knowledge in the areas of basic family health and some skills in diagnosis of acute childhood illness including respiratory illnesses and vitamin A deficiency. Supervision is not as regular as might be needed and is done by government village health workers. Refresher training is the major form of supervision [9]. The FCHVs now receive a small allowance for the days they attend training but work essentially as volunteers.

##### Impact

The FCHV programme has been attributed to making a substantial contribution to reducing infant and child morbidity and mortality. From 1986 to 1989, research was conducted in Jumla, a remote mountainous district in Nepal, which reported a 28% reduction in under-5 mortality through active case finding and management of pneumonia by trained paid community-based project workers using oral antibiotics [23]. This validated results from a previous study by Pandey et al. [24] showing a 59% ARI-specific mortality reduction with community-based treatment of childhood pneumonia. Following integration into the work of the FCHVs, a mixed method evaluation showed that 19% of acute respiratory infections were appropriately treated in health centres by trained health professionals compared to 35% by FCHVs [25,26]. Khanal et al. [27] found that FCHVs who made home visits to newborn babies in the first week of life were able to identify possible severe bacterial infections in 90% of cases and refer to facilities thereby reducing mortality rates from 5.3% to 1.5%. Shrestha [28] showed that empowerment-based training of FCHVs increased use of contraception from 0% to 52.3% among 241 married women.

##### The effects of the remuneration model

While the Female Community Health Volunteers were initially paid, remuneration was discontinued after a year due to lack of funds. This caused confusion in communities for many years as well as unfulfilled expectations among FCHVs. In a recent review of the programme, stakeholders expressed concern about the possibility of the introduction of a salary to the sustainability of the programme. They described that, “Whether you (government employee) work or not doesn’t matter. You go to the office, you get paid. But for the FCHV’s it’s a social obligation attached to their prestige…”. The perception that a health worker is being paid somehow creates a negative feeling in the Nepali society. The women in the programme expressed that the main motivator was to earn religious merit by serving their community; recognition by the community and improving health of the community were also mentioned. Other factors included acquisition of health knowledge, betterment of their family’s health, a change from routine work and social recognition. Others continue to hope for future benefits through direct financial incentives or employment. The women do receive gifts including bicycles, radios and saris [9] in addition to awards, certificates and ceremonies from the government and community.

Glenton et al. [29] suggest that the issue of monetary versus social markets is critical to the success of the programme, noting that monetary incentives to reward social effort can be detrimental. They state that:

“Differentiating between FCHV’s’ intrinsic and extrinsic motivations may not be useful as some of these motivations, such as the (intrinsic) desire to help others and the (extrinsic) social recognition this leads to, are strongly intertwined. Nevertheless, our study does illustrate that certain incentives have the potential to undermine others. The assumption that an individual’s motivation can be increased simply by increasing the amount of material incentives appears therefore to be wrong. Instead, we must understand the particular conditions that influence these motivations when selecting appropriate incentives.”

They note [29] that the “crowding theory” developed by Frey and Jegan in 2001, which states that extrinsic goals, coming from outside, can crowd out intrinsic goals where the person receives no apparent rewards except the activity itself, is relevant to volunteer CHWs. In this case, the intrinsic motivation received from serving the community may result from a feeling that the work is worthwhile or from the satisfaction gained from achieving goals [29].

#### Volunteers that sell health merchandise: Community Health Volunteer programme in Bangladesh

##### History and description

BRAC began in 1972 as a small relief organization. It has expanded its efforts to integrated sustainable development, poverty alleviation through microfinance and healthcare. Serving 110 million people in Bangladesh, it has 117 000 staff and 80 000 Community Health Volunteers (CHVs) and has now expanded its efforts to 10 other countries [30]. The CHVs, also known as Shasthya Shebikas, are generally 25 years of age or older, married with children not below 2 years, have had some years of schooling, willing to provide voluntary services and acceptable to the community they serve. They receive 3 weeks of training to distribute family planning methods, identify pregnant women, provide essential newborn care and postnatal visits, assist with deliveries, provide special care to low birth weight babies, manage diarrhoea and acute respiratory infections of children, oversee TB treatment, treat minor ailments and sell health commodities. They are then expected to provide home-based health education, treatment of basic health problems, health data collection, sell medicines and health commodities and make referrals to health centres as necessary [31].

According to UNICEF [9], BRAC has a strong monitoring and evaluation programme which it uses to identify weaknesses and correct them before expanding its efforts. BRAC provides supportive supervision with a programme organizer (PO) supervising 25–30 CHVs two to three times a month. Statistics from 2006 to 2008 have found attrition rates of 11.6% in rural areas and 15.5% in urban areas. This may, however, be higher in some places – with a 20% to 32% attrition rate reported in a study by Alam et al. [32]. Reported motivational factors for remaining in the programme vary. Women from often-marginalized communities gain community recognition and skills; however, the income generated is also important to the women. The incentive and income structure is based on three main approaches. The health workers are considered volunteers that can get a second loan from BRAC, sell health commodities to make a small profit and charge a small service fee for antenatal care. The average number of years women had been working in the 2011 [32] study was 5.8, with an average of 3.6 h per day spent in the field and 14 homes visited a day. Average income was $14 per month.

##### Impact

Little data on the impact of the CHVs on health outcomes is available. Alam et al. [32] note that the majority of the previous studies on CHVs do not provide a rigorous analysis regarding the extent to which different factors affect their performance. Well-trained CHVs were significantly better at identifying acute respiratory infections in children under 5 years of age in a case–control study of 120 volunteers [33]. Islam et al. [34] found that BRAC CHVs had as high a cure rate among patients with tuberculosis as government health workers (84% versus 82%) but for a lower cost (US$ 64 versus US$ 96).

##### The effects of the remuneration model

For the BRAC CHVs, the financial incentives are a strong source of motivation. Reichenbach and Shimul [31] note that 86% of the respondents in a study of the CHVs said they had become a CHV “to contribute to the income of their household”, with 3% stating “social recognition” and 1% reporting “helping her community”. In one study, it was noted that CHVs perceived themselves as “salesperson aiming to make a profit” instead of a volunteer worker [30]. Insufficient profit for their time and family members being unhappy with their work were the most frequently cited reasons for dropping out of the programme [9]. It seems therefore that funding arrangements can determine the way in which the CHW understands their purpose.

#### Volunteers with financial incentives: the Accredited Social Health Activist (ASHA) programme in India

##### History and description

The Accredited Social Health Activists (ASHAs) programme began in 2005 as a strategy under the National Rural Health Mission (NRHM) in India. The ASHAs are mostly females aged 25–45 with an 8th grade qualification [35]. According to Gopalan et al. [36] in 2011, there had been a total of 820 000 women that had been trained and deployed by 2011. They receive 23 days of training and are expected to attend weekly meetings at the local primary health centre with auxiliary nurse midwives. The ASHAs were envisioned to have three roles according to the National Institute of Health and Family Welfare in 2005. These were as follows: (1) service extenders to achieve government policy goals, (2) bridges between the rural people and the health service outlets and (3) social change agents to create awareness of the social determinants of health and mobilizing the community toward health planning and utilization of local health services [2]. Rather than a fixed stipend, the ASHAs receive performance-based payments for particular healthcare programmes, including the Janani Suraksha Yojana (JSY) programme, which provides a conditional cash transfer scheme to promote deliveries in institutions – $35 for the woman delivering and $15 for the ASHA who brings her to the health facility [37]. Additional payments might be given for promotion of immunization, family planning including sterilization for men and women, dispensing of TB medication, leprosy detection and referral and organization of village health nutrition days [38]. Paul et al. [37] note that the Indian primary healthcare system aimed to improve maternal and child health outcomes is challenged because of a “lack of capacity for training and supervision, lack of ownership…, lack of review and monitoring nationally and in states, inability to integrate the referral component and non-availability of line supervisors”.

##### Impact

India has not achieved coverage of more than 55% for any of the priority interventions for reproductive health and child health and nutrition. Forty-six per cent of infants were exclusively breastfed for the first 6 months of life in 2007–2008, and 24% received solid feeds and breastfeeds at 6–9 months [37]. There has, however, been an increase in facility-based deliveries of 8%, from 29.8% to 37.8% between 2002–2004 and 2007–2009 [39] in some areas where incentives for institutional-based deliveries are operating and a decline by 70% in neonatal mortality in areas with incentives linked to these targeted services [37-39]. The programme did not, however, seem to effectively reach the poorest of the poor [39]. Das et al. [40] suggest that the data from Lim et al. [39] misrepresents the success of the programme and that the percentage of women delivering at a health facility and receiving the incentive is lower than reported.

##### The effects of the remuneration model

There is conflicting information about the impact of incentives on the motivation of the CHWs in the ASHA programme. Gopalan [36] found that individual- and community-level factors, a sense of responsibility and feelings of self-efficacy were the main motivators for the 386 ASHAs interviewed in the state of Orissa. Others, however, suggest that financial remuneration is one of the key motivational factors [38] and that delays in payments are a disincentive for ASHAs and undermine their motivation to perform [41].

Nandan et al. [41] note that most ASHAs spend their time in areas linked to incentives and that many other tasks, such as health education, get less attention due to lack of incentives. Wang quoting Oxman and Fretheim in 2008 notes:

“Although performance based payment increases the quantity of health services delivered, it may also have unintended economic effects, such as distortion (neglect of important tasks that are not rewarded with financial incentives), gaming (improving or cheating on reporting rather than improving performance), corruption, cherry picking (serving easy-to-reach patients), widening the resource gap between the rich and poor, dependency on financial incentives, demoralization and bureaucratization” [38]*.*

Thirty-three per cent of the ASHAs feels that their income is not reasonable in view of their efforts, and the delay in the payment of incentives has undermined their motivation. Scott and Shanker [2] found that ASHAs were institutionally limited by (1) the outcome-based remuneration structure, (2) poor institutional support, (3) the rigid hierarchical structure of the health system and (4) a dearth of participation at the community level. They felt that the ASHAs were achieving partial success as service extenders [2] but that the incentive system was preventing them from being cultural mediators or broader social change agents.

#### Paid full-time CHWs – Behvarz programme in Iran

##### History and description

The Behvarz (CHW) programme in Iran began in 1979 [42]. The Behvarzs work at health houses – the most basic unit of the health system in Iran. The CHWs (one male and one female for each village) are selected by the communities, should have completed high school, been resident in the community for at least 1 year and be trained for a period of 2 years during which they receive accommodation and financial support. There are currently 31 000 Behvarzs in Iran – 1 per 1500 population [42]. University graduates are employed to provide training in three blocks during the 2 years. Funds come from the annual budget of the national and provincial health systems. The training has also recently been offered as a university course to improve standards. There are monthly refresher courses and supervisory visits from health centres. The Behvarzs are involved in multiple tasks including home visits for health promotion, immunization of children, breastfeeding, use of iodized salt, screening and follow-up of chronic illnesses and appropriate treatment for children suffering from diarrhoea and acute respiratory infections. They also ensure the rural population has access to vaccines, essential drugs and oral rehydration [43-46] and referral to an urban centre for specialized care [47]. In addition to a salary, there are additional incentives including training allowances, celebration of a “National Behvarz Day”, creation of the “excellent Behvarz award” and provision of personal loans [42,48,49].

##### Impact

Improvements in health indicators in Iran have been ascertained by census data; national health surveys which include questions about nutrition, child health and family planning; and death registries [43]. Iran has had dramatic improvements in their infant and maternal mortality rates that have been attributed, in part, to the Behvarz programme [43,49]. Mortality rates among infants and children younger than 5 years decreased from 93 and 135 deaths per 1000 live births in 1974 to 29 and 36 deaths per 1000 live births in 2000, respectively. In addition, the maternal mortality ratio, recognized as a sensitive indicator of both development and health services, decreased sharply from 140 to 24 deaths per 100 000 live births between 1985 and 2007. Life expectancy increased from 55.7 years in 1976 to 71.6 years in 2003. Specifically, improvement in management in diabetes and hypertension, reduced goitre and improved immunization and breastfeeding rates have been attributed to Behvarzs [42,45]. Of particular interest has been the narrowing of the gaps between the rural and urban areas [42].

##### The effects of the remuneration model

The Behvarz programme requires high levels of government commitment with payment as both students and when engaged full time in the community. The Behvarz then commits a minimum of 4 years to government service [42,48]. As might be expected, higher levels of mastery have been achieved in this type of programme where a minimum basic educational level is required and which provides higher quality training and meaningful supervision. In some ways, the programme in Iran, which offers two full years of training, has become more like an auxiliary or enrolled nurse programme than a CHW programme. The Behvarz programme includes 13 months of supervised clinical training [42]. The Ministry of Health has shown a high commitment to learning about the needs of the community and offering training in response to these.

#### Both full-time and part-time volunteer CHWs – Health Extension Worker programme in Ethiopia

##### History and description

The Health Extension Worker (HEW) programme began in 2003. The programme’s objectives are to provide “a package of basic and essential promotive, preventive and curative health services targeting households in a community, based on the principle of Primary Health Care (PHC) to improve the family’s health status with their full participation” [12]. Since January 2004, 38 000 HEWs have been trained. The HEWs, young local women with grade 10 education levels, are given 1 year training prior to employment with the woreda (district) health office. They work from a health post in their community, with a ratio of five health posts for every health centre. The HEW works with 5000 people and spends 75% of her time in the field. Alone, it has not been possible for the HEWs to reach the whole population, and the community health promotion programme is centred on volunteer community health promoters (vCHPs), working under the supervision and guidance of the HEWs and more recently the Health Development Armies (HDAs), who are members of model families [13]. The programme focuses on four major areas and provides 16 different packages, which the HEW, vCHPs and HDAs are trained to use with 50–60 model households over a 96-h period during 3 to 4 months on topics including the following: hygiene and environmental sanitation, family health services, disease prevention and control and health education and communication. Training is offered to any interested members of “model families” from teenagers to grandparents. Those that show interest or potential then become vCHPs who work with the HEWs to extend their reach and to train and collaborate in community action with other model families [13]. Through this combination of full-time and volunteer workers, much greater coverage can be achieved especially when they share the same goals.

##### Impact

In 2010, detailed reports were compiled analysing strengths and weaknesses of the HEW programme by the Centre for National Health Development in Ethiopia, the Earth Institute at Columbia University and the Ministry of Health in Ethiopia [13,14,50]. Early results from areas where HEWs, vCHPs and HDAs are deployed are encouraging: contraceptive use, clean water sources and pit latrines, HIV testing and antenatal visits all appear to be increasing. However, delivery, postnatal and outpatient health services were not sought from health posts with women preferring to have their babies at home [14,50]. In 1990, the under-5 mortality rate was one of the highest in the world at 204/1000 live births; by 2012, this rate had been reduced to 68/1000 live births [51]. Datiko and Lindtjørn [52] in a randomized controlled trial found that the involvement of HEWs improved case detection and treatment success for TB.

The HEW programme is still in a relatively early stage of development and appears to have an ongoing commitment to understanding the weaknesses of the HEWs and improving them. So far, the HEWs have achieved mastery in certain areas, including family planning and health and environmental education, with improvements in a number of health indicators. The government has shown a commitment to further improve their mastery in other areas, including diagnostics and treatment of common illnesses through improved training and supervision.

##### The effects of the remuneration model

The full-time HEW programme of Ethiopia has achieved a great deal of government support. Like the Behvarz programme, it has required considerable financial commitment from the government. This high level of commitment has translated into an ongoing learning process that has enabled the HEW programme to evolve according to the action and reflection process and the consequent learning that has taken place within the country. There was a recent realization that the HEWs may not be able to fulfil all of the health needs of the community. Through a process of consultation with the HEWs, it became evident that they were less content with their remuneration as they were required to fulfil more goals. In 2011, the majority (96.6%) of HEWs believed that they were underpaid considering their heavy workloads [12]. A response to this problem was to have greater involvement of vCHPs and members of model families, the Health Development Armies, who were then able to increase coverage as demands grew. This has shown some promising results with increasing levels of community engagement through this process.

## Discussion and evaluation

The purpose of this review is to understand broadly five remuneration models utilized by large-scale, well-established CHW training programmes in different parts of the world and to gain insight into their effect on the motivation and focus of the CHWs and how this might impact on government planning of CHW programmes.

### Motivation and focus

From the information available in the literature, it appears that – according to behavioural economics theory – CHWs work best within a social or monetary market rather than a mixed market [11,18,20]. A social market is one where volunteering one’s time for the common good, social status and community appreciation are the most important motivators whereas the monetary market is one in which the CHWs are trained and work full time and are remunerated for the hours expected of them. Because of the nature of the work, both groups benefit from a strong sense of service to their community or altruistic capital [20]. Both full-time and volunteer CHWs can become demotivated if they do not have access to adequate training, quality supervision, community acceptance or appreciation or if they are expected to work longer hours than they can realistically manage while fulfilling their other commitments. Full-time paid CHWs can further lose motivation if their allowances are not provided in a timely fashion.

The use of incentives or low remuneration, which falls into the category of a mixed market according to behavioural economics, such as in the ASHA programme in India, appears to create an environment where intrinsic motivation can be lost, focus is placed on incentives and discontent can occur. Time-consuming means to raise funds such as the sale of commodities can prevent part-time CHWs, who have many other responsibilities, from focusing on health issues. The use of gifts and community appreciation seems to be of value to all CHWs.

Remuneration models can also affect the health problems on which the CHW focuses, which is also important for planners to take into consideration. These case studies showed that the programmes that utilized volunteers or full-time paid CHWs or a combination of the two, successfully achieved community engagement with communities taking ownership for change. When planners are looking for health extenders [2] to fulfil existing government goals, incentives were effective. For example, when children needed to be mobilized for immunization or mothers escorted to health centres for antenatal visits or delivery, an incentive can be provided to a trained CHW who will help fulfil these goals. If, however, the goal is mixed – community engagement and incentivized health work – the focus of these CHWs is predominantly on areas where incentives exist with little or no time given to community empowerment. Financial incentives can also lend themselves to falsification, and the CHWs can become demotivated and discontent if they feel the funds are inadequate or there is a delay in them reaching the CHW.

### Categories

Categories of CHW remuneration differ according to the purpose of the CHW programme, the context in which it operates, commitment by the government and the funds available. Throughout the course of the case studies, it became increasingly evident that defining CHWs as “lay persons who have received some training to deliver healthcare services but are not professionals” [1] may no longer be adequate to encompass the range of roles being undertaken by this cadre of individuals. All of the programmes had high-retention rates and contributed, albeit in different ways, to improving health outcomes in the communities they were working. However, comparing the work of a Behvarz in Iran who has received 2 years of university-based training and is employed full-time in government service with a FCHV in Nepal who receives 15 days of training and works with the community 5 h per week does not seem useful. Despite the higher level of mastery that the Behvarzs achieved, the role of the FCHVs might be seen as equally important as they demonstrated high levels of community trust that government workers, at least in the Nepalese setting, may have failed to do. Having two broader categories – (1) CHWs that are trained to be part of the formal work force and paid according and (2) CHWs that receive minimal training and offer limited hours according to the context – may be more useful.

### Role of the government

Countries that have successfully managed to begin the long process of addressing their rural health needs have done so with high levels of government commitment and through a process of ongoing action, reflection and rethinking at every level. This is particularly evident in Ethiopia and Iran but is also seen in Nepal [1,12-14,48,49]. Whether paid or voluntary, the role of being a rural-based CHW is a demanding one, and selecting the right candidate who is community minded, resilient, can withstand challenges in the field and able to solve problems innovatively will be important whether paid or not. Presenting the programme in a way that builds the individual’s altruistic capital may be important for planners to consider. Equally, having a regular flow of funds to the CHWs if remunerated is important to avoid demotivation.

### Limitations

It was decided to include only one case study for each type in order to achieve some depth of understanding. This resulted in the loss of what could have been learned if we were able to include more examples. Comparison is difficult due to the diverse nature of the case studies. Even for sustained programmes, it was often difficult to find quality direct impact data on the effectiveness of the CHWs but rather showed improvement in overall health in that country. The challenges in defining a “CHW” have been discussed elsewhere in the paper.

## Conclusion

The case studies examined in this paper show that a number of different approaches to CHW training and remuneration can be successful. According to the type of outcomes intended for the CHW, and the context, planners might choose one type of remuneration over another. While the case studies focused on the developing world context, it can equally be applied in wealthier countries. Categorizing according to (1) CHWs that are trained to be part of the formal work force and paid accordingly and (2) CHWs that receive minimal training and offer limited hours according to the context, provides a means by which selection criteria, expectations, remuneration, training, roles and expectations can more easily be delineated without losing the benefits of either approach.

The case studies show that the issue of payment is more complex than might initially appear and can undermine the programme if not thought through well. If payment or incentives are perceived as inadequate or do not flow in a timely fashion, then this can be demotivating to the CHW. Part-time models should not place unrealistic expectations on the CHW’s time or capacity. Maximizing community appreciation and altruistic capital within the programme is also important for planners. Other models will no doubt emerge, and quality research is needed to understand the relationship between remuneration and motivation. Remuneration does not, however, stand alone, and ongoing research will be needed to understand the complex issues of community engagement, CHW appreciation, meaningful supervision and the relationship and need for paid and unpaid human resources in a system that can empower the communities to improve health. Governments and planners have an opportunity to examine the most cost effective and sustainable models that can increase community engagement and thereby reduce the overall healthcare costs.

## Abbreviations

CHWs: Community health workers
LMIC: Low- and middle-income countries
FCHV: Female Community Health Volunteer
CHV: Community Health Volunteer
ASHAs: Accredited Social Health Activists
NRHM: National Rural Health Mission
vCHPs: Volunteer community health promoters
HDAs: Health Development Armies
